# *Lacticaseibacillus paracasei* FJG2337 mitigate acute liver injury-related inflammatory responses, gut microbiota and liver metabolism in mice

**DOI:** 10.3389/fcimb.2025.1674551

**Published:** 2025-10-21

**Authors:** Siyu He, Yuheng Yang, Weiling Guo, Rongwen Ou, Xucong Lv, Youting Chen

**Affiliations:** ^1^ Department of Hepatopancreatobiliary Surgery, The First Affiliated Hospital of Fujian Medical University, Fuzhou, China; ^2^ Institute of Food Science and Technology, College of Biological Science and Engineering, Fuzhou University, Fuzhou, Fujian, China; ^3^ Fujian Abdominal Surgery Research Institute, The First Affiliated Hospital, Fujian Medical University, Fuzhou, China; ^4^ Department of Hepatopancreatobiliary Surgery, National Regional Medical Center, Binhai Campus of the First Affiliated Hospital, Fujian Medical University, Fuzhou, China; ^5^ Clinical Research Center for Hepatobiliary Pancreatic and Gastrointestinal Malignant Tumors Precise Treatment of Fujian Province, The First Affiliated Hospital, Fujian Medical University, Fuzhou, China

**Keywords:** *Lacticaseibacillus paracasei*, inflammatory responses, oxidative stress, gut microbiota, liver metabolism

## Abstract

Acute liver injury (ALI) has a high global prevalence but limited and suboptimal treatment options. Probiotics can help improve liver function by regulating gut microbiota, suppressing inflammatory responses, and inhibiting oxidative stress. However, substantial variations exist in the hepatoprotective efficacy of different bacterial strains. *Lacticaseibacillus paracasei* FJG2337 was isolated from the feces of long-lived elderly individuals, but its potential hepatoprotective effects remain unclear. In the present study, the influence of *L. paracasei* FJG2337 on the liver function in lipopolysaccharide (LPS)-treated mice was investigated. Our data demonstrated that *L. paracasei* FJG2337 ameliorated the serum parameters (including ALT, AST, and ALP) and elevated cecal short-chain fatty acids contents in mice with ALI. *L. paracasei* FJG2337 pre-treatment suppressed inflammatory responses and oxidative stress in ALI mice through modulating the TLR4/NF-кB and Nrf2/HO-1 pathway, respectively. Furthermore, *L. paracasei* FJG2337 significantly elevated the abundance of beneficial bacteria in mice and significantly reduced the harmful bacteria populations. Metabolomics analysis displayed that *L. paracasei* FJG2337 ameliorated liver metabolism in ALI mice, primarily affecting tryptophan metabolism, arachidonic acid metabolism, retinol metabolism, and glutathione metabolism, and so on. These results highlight that *L. paracasei* FJG2337 could serve as a promising probiotic intervention for patients with liver function injury.

## 1 Introduction

The liver is the primary organ responsible for the energy and drug metabolism, as well as the regulation of detoxification. Over the past few decades, alteration in lifestyle and eating habits have resulted to a gradual increase in the number of patient with liver disease (Gu et al., 2025). Among them, acute liver injury (ALI, known as acute liver failure) has emerged as a major global public health concern because of it’s the relative high morbidity and mortality (Torgersen et al., 2021). Previous investigations have shown that the onset and progression of ALI can be triggered by various factors, including endotoxins, viral infections, radioactive substances, and drug toxicity. These cases typically present with abnormal serum levels of liver enzymes [alanine transaminase (ALT) and aspartate transaminase (AST)], along with oxidative stress and inflammatory responses (Guo et al., 2025). Although the molecular mechanisms of ALI involve multiple pathways, oxidative stress and inflammatory responses are recognized as critical factors that accelerate both the onset and progression of ALI. Lipopolysaccharide (LPS), a harmful component predominantly found in gram-negative bacterial cell walls, promotes the production of pro-inflammatory cytokines while simultaneously impairing antioxidant enzyme activity (Guo et al., 2021). The ALI model was successfully established using LPS, which serves as a representative and widely utilized model for studying the hepatoprotective effects of therapeutic agents. At present, anti-inflammatory drugs and liver transplantation are commonly employed to manage ALI, but drug-related side effects and the limited availability of donor livers often delay optimal treatment. Consequently, identifying functional dietary supplements to ameliorate ALI has become a critical priority in public health research.

Probiotics are live microorganisms that serve as essential components of human microecosystem, which play the most important role in improving host health, such as the enhancement of immunity, amelioration of inflammatory bowel disease, regulation of gut microbiota (GM), hypoglycemia, and hypolipidemia (Wang et al., 2025). Among them, *Lactobacillus* and *Bifidobacterium* are the most prevailing probiotics that frequently used in both fermented products and medicinal applications, especially *Lactobacillus*. *Lactobacillus* is a facultative anaerobe that has been shown to suppress inflammatory responses, enhance antioxidant enzymes activity, as well as regulate the GM composition. For instance, oral administration of *L. plantarum* KFY02 prevented the accumulation of pro-inflammatory cytokines [including tumor necrosis factor-alpha (TNF-α), interleukin-6 (IL-6), nterleukin-12 (IL-12), and interferon-gamma (IFN-γ)] and elevated the activity of antioxidant enzymes [including superoxide dismutase (SOD) and glutathione peroxidase (GSH-Px)] in mice with liver damage (Pan et al., 2020). *L. paracasei* S16 intervention also reduced the accumulation of pro-inflammatory cytokines and regulate the differentiation of T cells (Wang et al., 2021). Our previous study found that *L. paracasei* CCFM1222 and CCFM1223 (from the feces of an infant) suppressed the product of pro-inflammatory cytokines and enhanced the activity of antioxidant enzymes in ALI mice, but their mechanisms of improvement differed significantly (Guo et al., 2023, 2022). Our preliminary experiments displayed that *L. paracasei* FJG2337, isolated from the feces of long-lived elderly individuals, exhibited potent free radical scavenging activity (**Supplementary Figure S1**), suggesting its potential as a probiotic strain. However, the impact of *L. paracasei* FJG2337 on inflammatory responses, oxidative stress, GM and liver metabolism in ALI mice has not been explored.

The present study aimed to investigate the potential ameliorative property of *L. paracasei* FJG2337 in alleviating inflammatory responses and oxidative stress in LPS-induced ALI mice, while elucidating its underlying mechanisms. Thus, the strain’s anti-inflammatory and antioxidant effects were evaluated through analysis of physicochemical parameters, gene expression profiles, and histopathological examination. Additionally, the high throughput sequencing and liver metabolomics were applied to demonstrate the potential hepatoprotective mechanisms of *L. paracasei* FJG2337. These results elaborate the potential hepatoprotective effects of *L. paracasei* FJG2337 and provide theoretical basis for its application as functional foods and nutritional supplements.

## 2 Materials and methods

### 2.1 Bacterial strain and materials

*L. paracasei* FJG2337 was previously isolated from the feces of long-lived elderly individuals. LPS, acetonitrile, and formic acid were purchased from Sigma-Aldrich (St. Louis, MO, U.S.A.). Commercial kits for measuring alanine aminotransferase (ALT), aspartate aminotransferase (AST), alkaline phosphatase (ALP), malondialdehyde (MDA), superoxide dismutase (SOD), glutathione peroxidase (GSH-Px), and catalase (CAT) were obtained from the JianCheng Bioengineering Institute (Nanjing, China). ELISA kits (TNF-α, IL-1β, IL-6, and IL-10) were supplied by Meimian Industrial Co., Ltd. (Shanghai, China).

### 2.2 Animals and experimental design

24 male C57BL/6J mice aged six weeks were purchased from Xingyi (Fuzhou) biotechnology Co. Ltd. (No. B202504070924) and kept in the standard conditions (22 ± 2°C, 55% relative humidity, and a 12 h light-dark cycle). After a one-week acclimatization period, the mice were classified into three groups: the control, model, and FJG2337 groups. Among them, mice in the control and model groups received 0.2 mL normal saline every day, while mice in the FJG2337 group were administered the equal volume of *L. paracasei* FJG2337 suspension (10^9^ CFU). Body weight was surveyed every two days during the whole experiment. After 14 days of intervention, fecal samples were collected and placed at -60°C, and then all mice were fasted for 4 h. The ALI mice in model and FJG2337 groups were built according to our previous method. All mice were euthanized using carbon dioxide asphyxiation followed by cervical dislocation after 10 h of fasting, blood, liver, spleen, cecal contents, and colonic contents were collected. All procedures involving animals in compliance with the guidelines granted by the animal ethics committee of the institute of food science and technology (Fuzhou, China, No. FZU-FST-2025-101).

### 2.3 Biochemical analysis

Blood samples were allowed to coagulate at 25°C for 2.5 h, and serum was collected after centrifugation (3,000 × g, 15 min, 20°C). Serum AST, ALT, and ALP levels were measured using commercial kits. Partial liver tissue was homogenized with normal saline at a 1:9 (w/v) ratio using a tissue homogenizer (Xinzhi Bio. Ltd. Ningbo, China). The homogenate was centrifuged (10,000 × g, 15 min, 4°C) to remove sediments, and the supernatant was collected for analysis. The supernatant TNF-α, IL-1β, IL-6, IL-10, MDA, SOD, GSH-Px, and CAT levels were measured using commercial available assay kits.

### 2.4 Histopathological analysis

Partial liver samples were fixed using 4% paraformaldehyde, embedded in paraffin, sectioned (3 μm), and stained with hematoxylin and eosin (H&E) by Servicebio Technology Co., Ltd (Fuzhou, China). Histopathological evaluation was performed using a digital slide scanner, and images were recorded for analysis.

### 2.5 Detection of mRNA expression of genes-ALI

Hepatic RNA was obtained using TRIzol reagent (Sangon, Shanghai, China), and its quality were measured by nucleic acid quantizer. Hepatic RNA was reverse-transcribed into cDNA using a commercial reverse transcription kit (Takala, Dalian, China), and then the mRNA expression of ALI-related genes was detected using CFX Connect Real-Time System (Bio-Rad, USA), with relative expression calculated via the 2^−ΔΔCt^ method. All primer sequences used in this study are listed in **Supplementary Table S1**.

### 2.6 Short-chain fatty acids analysis

Cecal contents from all mice were collected, freeze-dried, and weighted before being transferred to centrifuge tubes (containing three steel beads and 0.5 mL of saturated NaCl solution). The samples were homogenized using organizational disruptor. Subsequently, 10 μL of dilute sulfuric acid was added to the homogenate, followed by centrifugation (10,000 × g, 15 min, 4°C) to remove particulate matter. The resulting supernatant was mixed with 0.8 mL of diethyl ether, and the upper organic phase was collected into an injection vial. Short-chain fatty acid (SCFA) concentrations were quantified by gas chromatography.

### 2.7 16S rRNA sequencing analysis

Total DNA from feces samples was obtained by a genome extraction kit (Magorbio, Shanghai, China), and then the V3-V4 region of 16S rDNA were amplified sing universal primers and purified by 2.0% agarose gel electrophoresis. The concentration of DNA from each sample was measured, and equimolar amounts of each sample were mixed to establish DNA libraries. Library quality was detected before paired-end sequencing on the Illumina Miseq PE300 platform. Raw sequencing data were processed using QIIME 2 (version 2020.6). Low-quality reads (quality score <20 or length <99% of expected size) were filtered out, and then annotated as the same operational taxonomic unit according to the similarity greater than 97%. Alpha and beta- diversity of GM was analyze using X shell software (v 8.0), the difference GM from different groups was screened by Wilcoxon rank-sum test.

### 2.8 Liver metabolomics

Liver tissue samples (30 mg) were homogenized with three steel beads in 0.8 mL of extraction solution (methanol:acetonitrile = 1:4, v/v) using a tissue homogenizer. After centrifugation (10,000 × g, 20 min, 2°C), the supernatant was collected and freeze-dried. The pellet was re-extracted with fresh extraction solution (0.5 mL) and centrifuged again (10,000 × g, 15 min, 4°C). For quality control, 20 μL aliquots from each sample were pooled. Metabolite profiling of each sample was detected by ultra-performance liquid chromatography-quadrupole time-of-flight mass spectrometry (UPLC-QTOF/MS) system, and the raw data were processed using CD software (v 3.2), including peak detection, alignment, and normalization. The overall composition of liver metabolites from difference groups were conducted by principal component analysis (PCA) and partial least-squares discriminate analysis (PLS-DA), and the potential metabolic features were screened by volcano plot. The enriched pathways of the potential metabolic features were analyze using commercial databases (http://www.kegg.jp).

### 2.9 Statistical analysis

All the data were displayed as the mean ± SD by GraphPad Prism software (v 9.0). The statistical differences among three groups were analyze by Student’s t-test, one-way analysis of variance (ANOVA) using SPSS software (v 22.0). ^*^ and ^**^ means *p* < 0.05 and *p* < 0.01, respectively.

## 3 Results

### 3.1 Effect of *L. paracasei* FJG2337 on body weight, organ indexes, and serum biochemical indicators in mice

**Figure 1:**
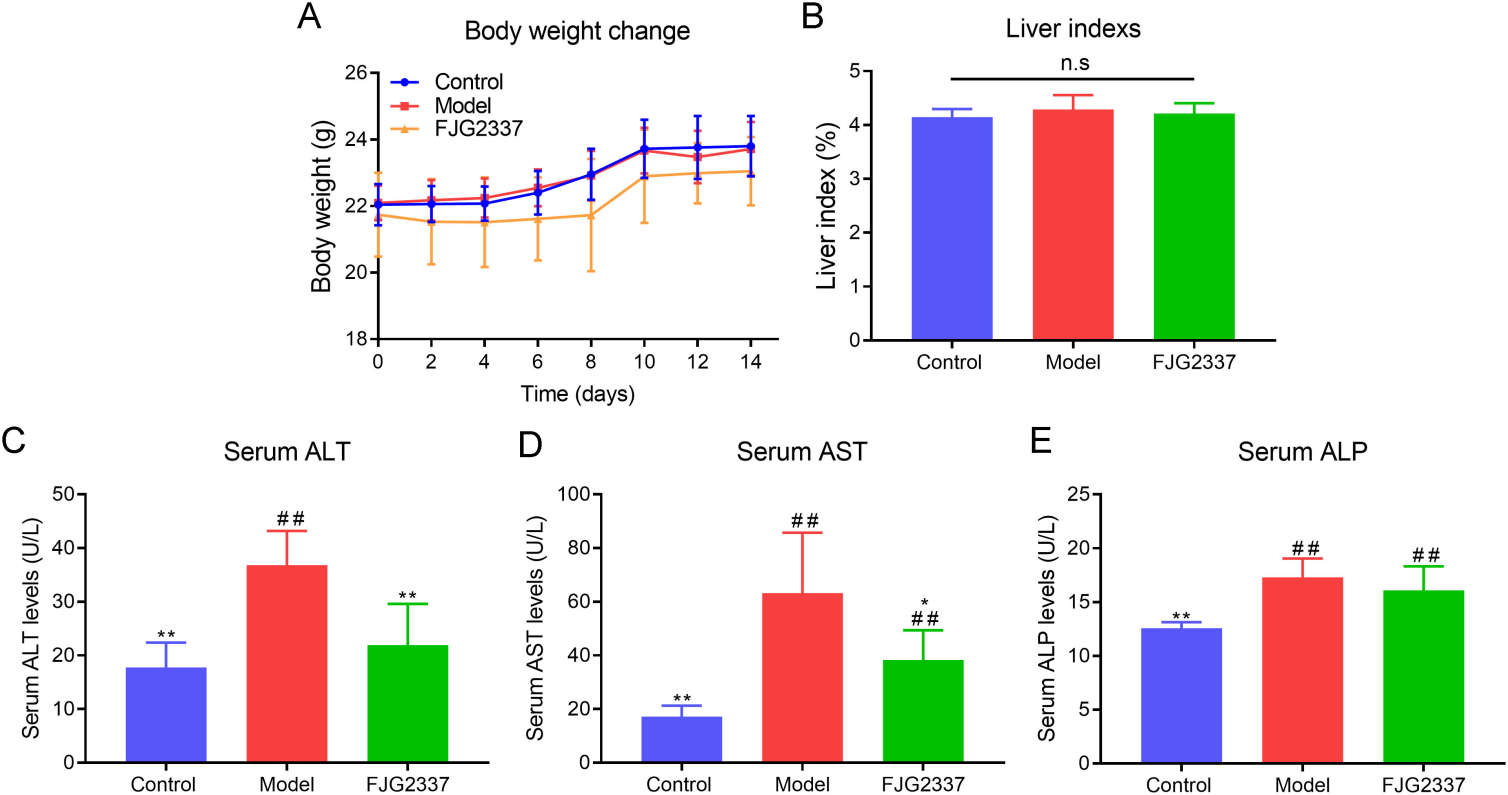
Impact of *L. paracasei* FJG2337 pre-treatment on body weight **(A)**, liver index **(B)**, serum AST **(C)**, serum ALT **(D)**, and serum ALP **(E)** on mice from the three group. Asterisk imply significant differences in relative to model group (^*^ *p* < 0.05 and ^**^ *p* < 0.01), while hashtag imply significant differences in relative to control group (^##^ *p* < 0.01) n.s. represents no significance.

As exhibited in **Figure 1A**, all mice exhibited gradual weight gain throughout the experimental period, with no significant differences observed among the three groups (*p* > 0.05), indicating that *L. paracasei* FJG2337 administration had no adverse effects on growth. Liver weight, which reflects the degree of hepatomegaly - a common clinical manifestation of acute liver injury (ALI)-showed a significant increase in the model group compared to controls (**Figure 1B**). This demonstrates that LPS challenge successfully induced hepatic enlargement. However, *L. paracasei* FJG2337 pre-treatment suppressed the enlargement of the liver in LPS-treated mice, implying its protective effect against liver enlargement. Furthermore, analysis of blood lipid parameters (TG, TC, HDL-C, and LDL-C) revealed no significant differences between the model and FJG2337 groups (*p* > 0.05, **Supplementary Figure S2**), suggesting that short-time consumption of *L. paracasei* FJG2337 did not significantly alter lipid metabolism dysregulation. Serum AST (63.24 ± 22.43 U/L *Vs*. 17.20 ± 4.08 U/L), ALT (36.80 ± 6.38 U/L *Vs*. 17.78 ± 4.62 U/L), and ALP (17.31 ± 1.74 U/L *Vs*. 12.56 ± 0.57 U/L) concentration were dramatically elevated in the model group compared to the control group (*p* < 0.05), suggesting that LPS could induce the liver function injury (**Figures 1C–E**). Importantly, *L. paracasei* FJG2337 pre-treatment significantly reduced the serum AST (38.33 ± 11.03 U/L *Vs*. 63.24 ± 22.43 U/L) and ALT (21.94 ± 7.65 U/L *Vs*. 36.80 ± 6.38 U/L) levels in ALI mice (*p* < 0.05), but it had no significant effect on ALP levels (13.09 ± 2.23 U/L *Vs*. 17.31 ± 1.74 U/L, *p* > 0.05). Interestingly, there was no remarkable difference in serum ALT (17.78 ± 4.62 U/L *Vs*. 21.94 ± 7.65 U/L) level between the control and FJG2337 group, but remarkable difference in serum AST (17.20 ± 4.08 U/L *Vs*. 38.33 ± 11.03 U/L) level. These results collectively suggest that *L. paracasei* FJG2337 shows potential for ameliorating LPS-induced liver function impairment, particularly in reducing hepatocellular damage as indicated by transaminase levels.

### 3.2 *L. paracasei* FJG2337 alleviates oxidative inflammatory response in ALI mice

**Figure 2:**
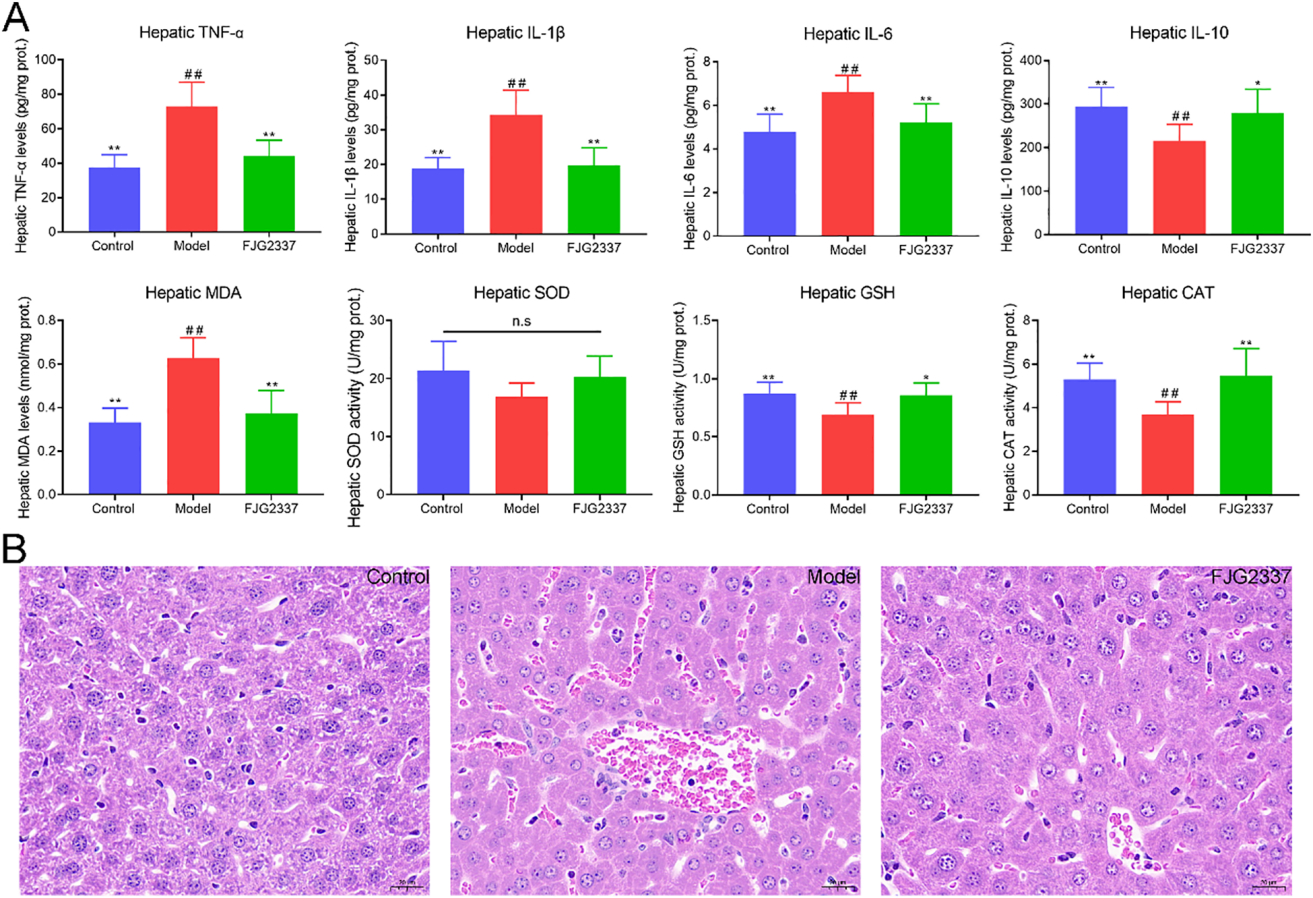
*L. paracasei* FJG2337 pre-treatment suppressed inflammatory responses, oxidative stress and liver injury in HFD-fed mice. **(A)** Hepatic inflammatory cytokines and anti-oxidative stress; **(B)** Representative images of liver pathology (scale bar: 20 μm, black arrows indicate areas of inflammatory cell infiltration, 20 × magnification). Asterisk imply significant differences in relative to model group (^*^ *p* < 0.05 and ^**^ *p* < 0.01), while hashtag imply significant differences in relative to control group (^##^ *p* < 0.01) n.s. represents no significance.

Intraperitoneal LPS administration disrupted the balance of oxidative and inflammatory responses. It can be obtained from **Figure 2A**, hepatic pro-inflammatory cytokine levels (TNF-α [73.05 ± 13.96 pg/mg prot. *Vs*. 37.46 ± 7.57 pg/mg prot.], IL-1β [34.34 ± 7.05 pg/mg prot. *Vs*. 18.85 ± 3.19 pg/mg prot.], and IL-6 [6.60 ± 0.77 pg/mg prot. *Vs*. 4.79 ± 0.81 pg/mg prot.]) were dramatically increased in the model group compared to the control group (*p* < 0.01), while the anti-inflammatory cytokine IL-10 were dramatically decreased (215.39 ± 38.05 pg/mg prot. *Vs*. 294.58 ± 43.47 pg/mg prot., *p* < 0.01). *L. paracasei* FJG2337 pre-treatment dramatically attenuated these LPS-induced effects, reducing pro-inflammatory cytokine levels (TNF-α [44.20 ± 9.21 pg/mg prot. *Vs*. 73.05 ± 13.96 pg/mg prot.], IL-1β [19.85 ± 4.95 pg/mg prot. *Vs*. 34.34 ± 7.05 pg/mg prot.], and IL-6 [5.22 ± 0.85 pg/mg prot. *Vs*. 6.60 ± 0.77 pg/mg prot.], *p* < 0.01) while increasing IL-10 production (279.66 ± 54.23 pg/mg prot. *Vs*. 215.39 ± 38.05 pg/mg prot., *p* < 0.05), demonstrating its anti-inflammatory properties. In addition, the hepatic MDA level was dramatically elevated in the model group compared to the control group (0.63 ± 0.09 nmol/mg prot. *Vs*. 0.33 ± 0.06 nmol/mg prot., *p* < 0.01), and the hepatic GSH (0.69 ± 0.10 U/mg prot. *Vs*. 0.87 ± 0.10 U/mg prot.) and CAT (3.70 ± 0.58 U/mg prot. *Vs*. 5.30 ± 0.75 U/mg prot.) levels were significantly reduced (*p* < 0.01). *L. paracasei* FJG2337 pre-treatment significantly decreased the hepatic MDA levels in ALI mice (0.37 ± 0.10 nmol/mg prot. *Vs*. 0.63 ± 0.09 nmol/mg prot., *p* < 0.01), and enhanced the hepatic GSH (0.86 ± 0.11 U/mg prot. *Vs*. 0.69 ± 0.10 U/mg prot.) and CAT (5.46 ± 1.25 U/mg prot. *Vs*. 3.70 ± 0.58 U/mg prot.) activity (*p* < 0.05). No significant differences in these parameters-related inflammation and oxidative stress between the control and FJG 2337 groups (*p* > 0.05).

Histopathological analysis revealed distinct morphological differences among the experimental groups (**Figure 2B**). Liver sections from control mice displayed normal hepatic architecture, with hepatocytes radially arranged around central veins and no observable pathological changes. In contrast, the model group exhibited characteristic features of acute liver injury, including significant inflammatory cell infiltration, widespread hepatocyte necrosis, and nuclear pyknosis. Notably, mice pretreated with *L. paracasei* FJG2337 exhibited marked improvement in hepatic histopathology, with reduced inflammatory infiltration compared to the model group, although some residual pathological features remained. The above results suggested *L. paracasei* FJG2337 pretreatment effectively ameliorated LPS-induced hepatic damage and ameliorates liver inflammation.

### 3.3 *L. paracasei* FJG2337 elevates the cecal SCFAs levels in ALI mice

**Figure 3:**
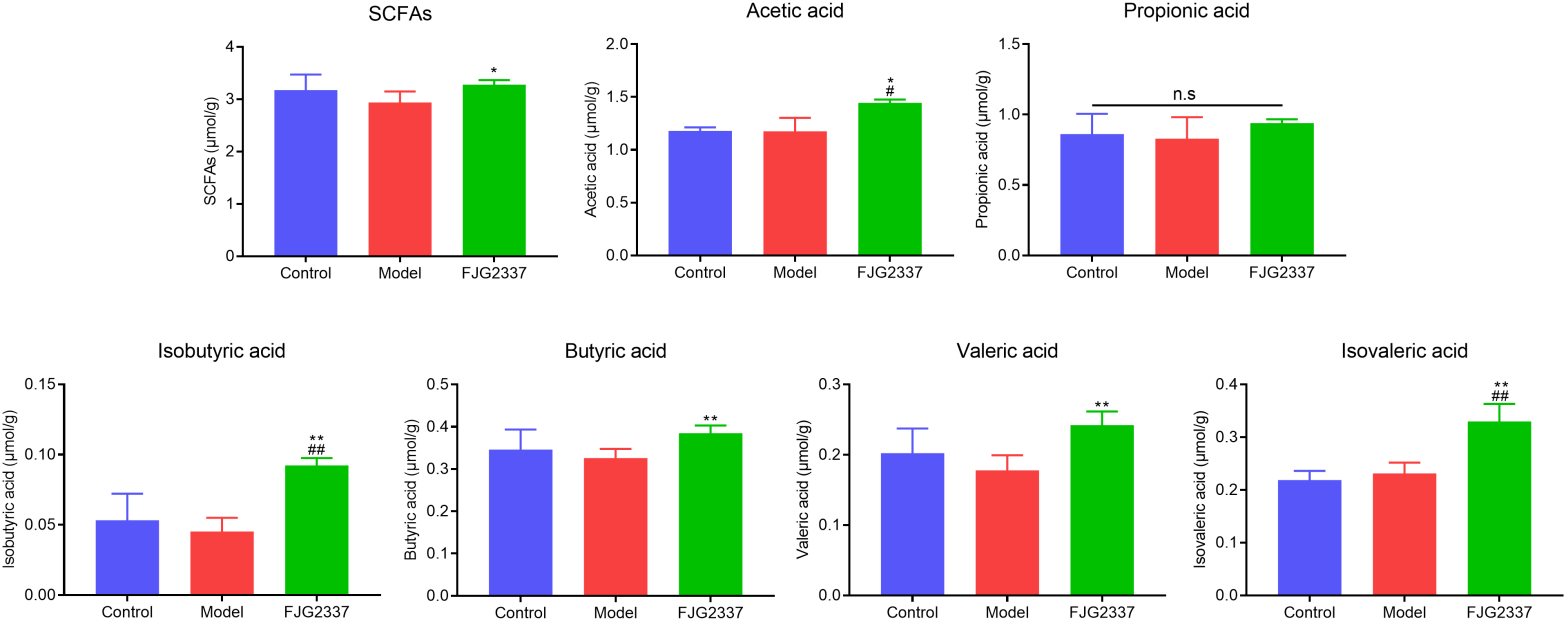
Impact of *L. paracasei* FJG2337 pre-treatment on cecal SCFAs levels in ALI mice. Asterisk imply significant differences in relative to model group (^*^ *p* < 0.05 and ^**^ *p* < 0.01), while hashtag imply significant differences in relative to control group (^#^ *p* < 0.05 and ^##^ *p* < 0.01) n.s. represents no significance.

SCFAs, as key microbial metabolites, play a pivotal role in gut-liver axis communication. As shown in **Figure 3**, no dramatically discrepancy in cecal acetic acid, propionic acid, butyric acid, iso-butyric acid and SCFAs between the control and model groups (*p* > 0.05). *L. paracasei* FJG2337 pre-treatment dramatically increased the cecal acetic acid, butyric acid, iso-butyric acid and SCFAs in ALI mice induced by intraperitoneal injection of LPS (*p* < 0.05), while propionic acid levels remained unchanged (*p* > 0.05). Compared with the control group, *L. paracasei* FJG2337 pre-treatment dramatically increased the cecal acetic acid and iso-butyric acid level (*p* < 0.05).

### 3.4 *L. paracasei* FJG2337 regulates the mRNA expression of genes-related ALI in LPS-treated mice

**Figure 4:**
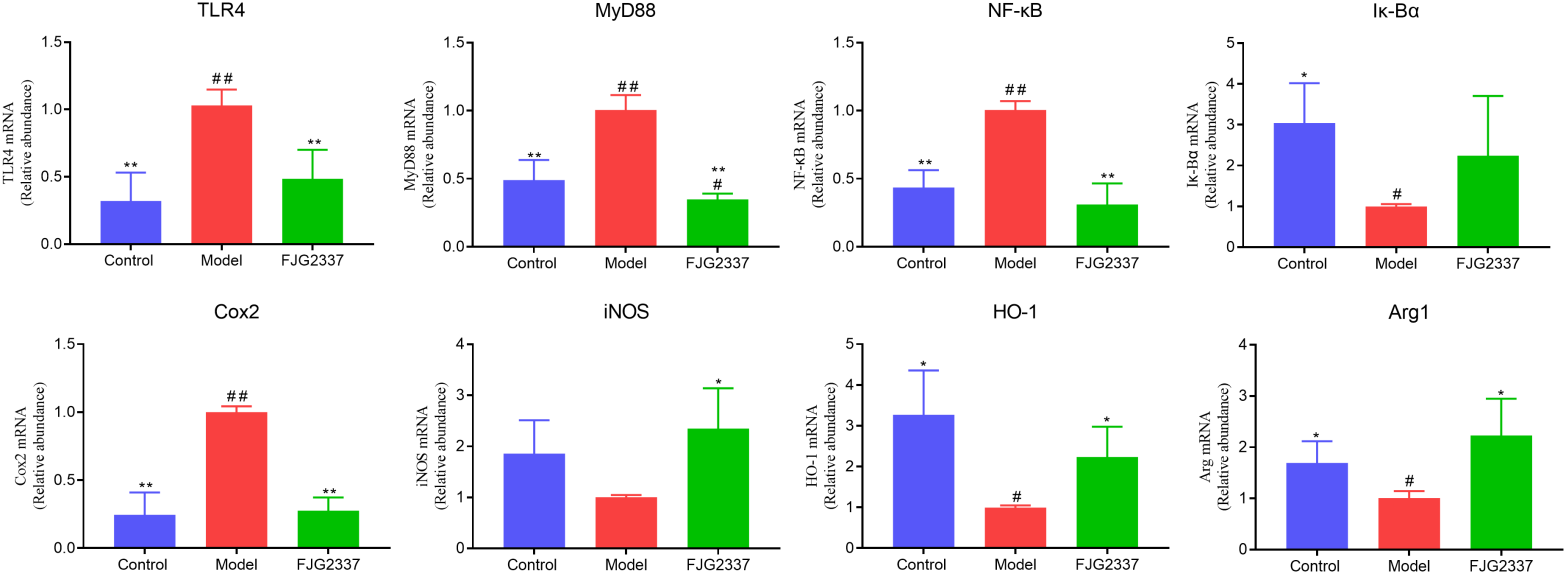
Impact of *L. paracasei* FJG2337 pre-treatment on the transcription level of genes-related inflammatory responses and oxidative stress in ALI mice. Asterisk imply significant differences in relative to model group (^*^ *p* < 0.05 and ^**^ *p* < 0.01), while hashtag imply significant differences in relative to control group (^#^ *p* < 0.05 and ^##^ *p* < 0.01).

To investigate the improvement effects of *L. paracasei* FJG2337 against LPS-induced ALI, hepatic gene expression profiles related to inflammatory responses and oxidative stress were measured. As depicted in **Figure 4**, LPS treatment remarkably upregulated the transcription level of TLR4, MyD88, NF-кB, COX2, and iNOS in the liver (*p* < 0.05), while down-regulated the transcription level of Iк-Bα, HO-1, and Arg1 (*p* < 0.05). Interestingly, *L. paracasei* FJG2337 pre-treatment dramatically attenuated these LPS-induced transcriptional alterations (*p* < 0.05), suggesting its hepatoprotective mechanism involves modulation of both inflammatory and antioxidant pathways. In addition, no remarkable difference in the transcription level of TLR4, NF-кB, COX2, iNOS, Iк-Bα, HO-1, and Arg1 between the control and FJG2337 group (*p* < 0.05).

### 3.5 *L. paracasei* FJG2337 regulates the GM composition in ALI mice

**Figure 5:**
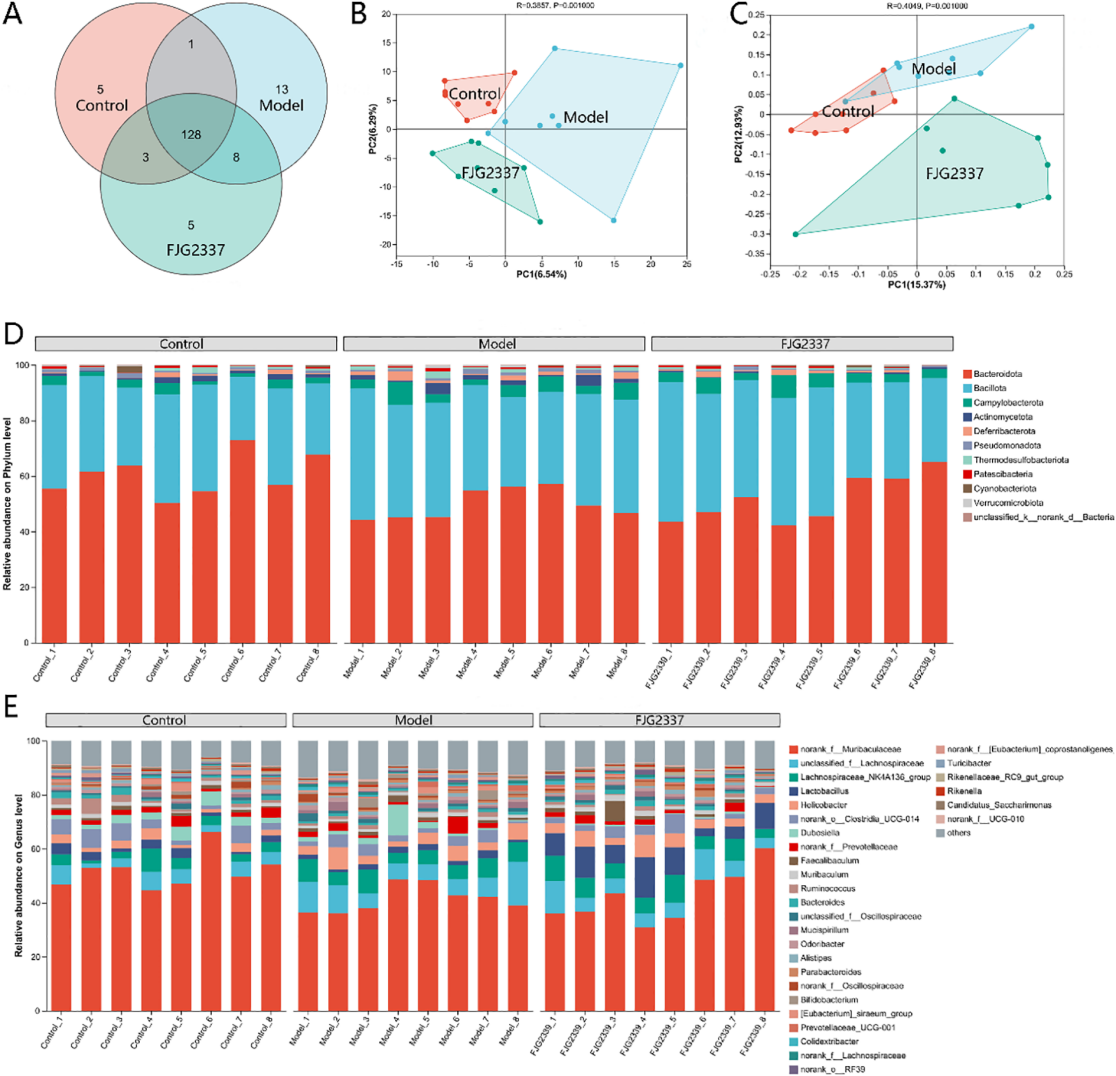
*L. paracasei* FJG2337 pre-treatment regulated the gut microbiota composition in ALI mice. **(A)** Venn diagram; **(B)** PCA analysis; **(C)** PCoA analysis; **(D)** Relative abundance of gut microbiota at the phylum level; **(E)** Relative abundance of gut microbiota at the genus level.

Venn diagrams are frequently employed to visualize shared and unique bacterial genera across experimental groups. The Venn diagram result exhibited a total of 163 genera common to all three groups, with the control, model, and FJG2337 groups exhibiting 5, 13, and 5 unique genera, respectively (**Figure 5A**). Multivariate analyses demonstrated significant microbial community differences among groups. The results of PCA displayed that an obvious separation among three groups, suggesting that *L. paracasei* FJG2337 effectively regulated the GM composition in LPS- challenged mice (**Figure 5B**). This finding was corroborated by the result of PCoA (**Figure 5C**). At the phylum level, the dominant bacterial taxa across all groups were Bacteroidota, Bacillota, Campylobacterota, Actinomycetota, and Deferribacterota, with Bacteroidota and Bacillota being particularly abundant (**Figure 5D**). Genus-level analysis identified *norank_f_Muribaculaceae*, *unclassified_f_Lachnospiraceae*, *Lachnospiraceae_NK4A136_group*, and *Lactobacillus* as the predominant taxa (**Figure 5E**).

**Figure 6:**
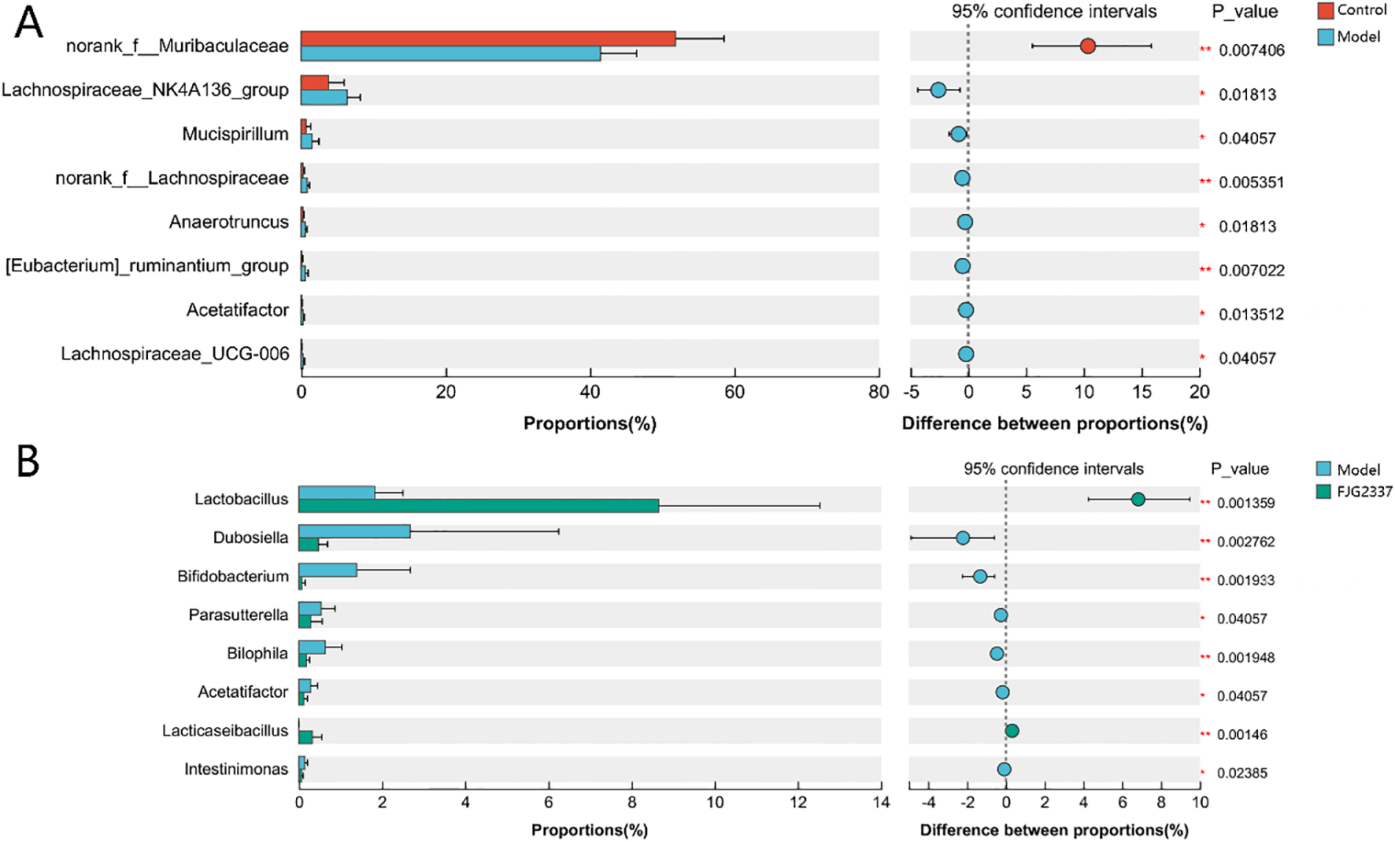
Wilcoxon rank-sum test bar plot on microbiota composition at the genus level. **(A)** control *Vs* model; **(B)** FJG2337 *Vs* model. Asterisk imply significant differences between Control/FJG2337 and Model groups (*p < 0.05 and **p < 0.01).

To identify significant alterations in GM composition among groups, we performed differential abundance analysis using the Wilcoxon rank-sum test. Compared with the control group, the proportion of *norank_f_Muribaculaceae* were dramatically reduced in the model group, while the proportion of *Lachnospiraceae*_*NK4A136*_*group*, *Mucispirllum*, *norank*_*f*_*Lachnospiraceae*, *Anaerotruncus*, [*Eubacterium*] *ruminantium*_*group*, *Acetatifactor*, and *Lachnospirac*eae_UCG-006 (**Figure 6A**; **Supplementary Table S2**). Notably, *L. paracasei* FJG2337 pre-treatment dramatically increased the proportion of *Lactobacillus* and *Lacticaseibacillus* in LPS-treated mice, but significantly reduced the relative abundance of *Dubosiella*, *Bifidobacterium*, *Parasutterella*, *Bilophila*, *Acetatifactor*, and *intestinimonas* (**Figure 6B**).

### 3.6 *L. paracasei* FJG2337 reversed the influences of LPS on liver metabolomics

**Figure 7:**
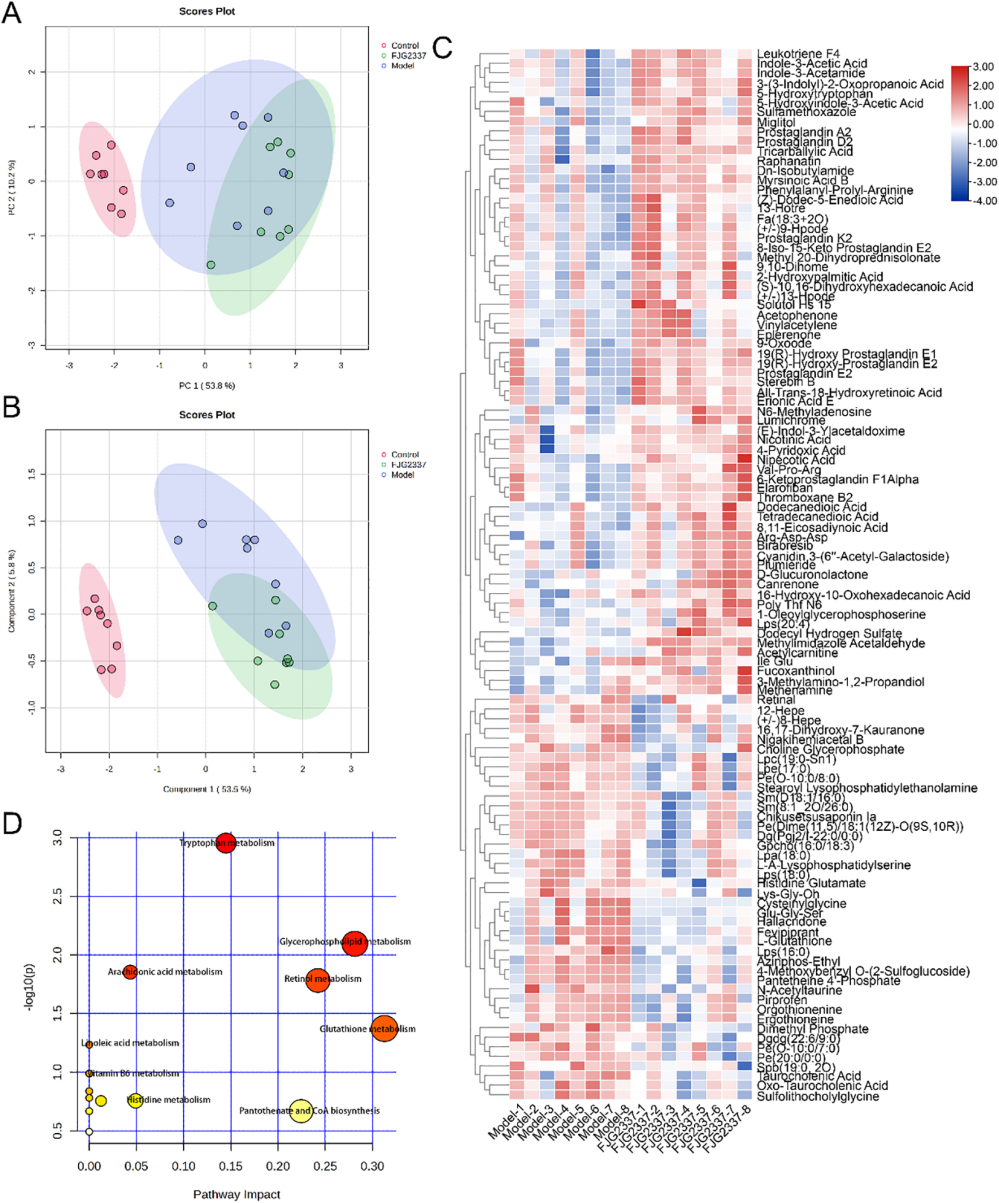
*L. paracasei* FJG2337 pre-treatment improve the liver metabolism in ALI mice. **(A)** PCA analysis; **(B)** OLPS-DA analysis; **(C)** Heatmap of significant difference in liver metabolites between the Model and FJG2337 groups; **(D)** Pathway enrichment analysis was carried out based on difference liver metabolites.

To comprehensively characterize the metabolic alterations in ALI mice following *L. paracasei FJG2337* pretreatment, untargeted liver metabolomics based on UPLC-QTOF/MS were performed in this study. In the PCA score plot, the distinct clustering patterns among the control, model, and FJG2337 groups, demonstrating significant metabolic perturbations in LPS-induced ALI mice (**Figure 7A**). The observed separation between groups indicates substantial changes in endogenous small molecule metabolites. This metabolic discrimination was further confirmed by the result of OPLS-DA, which showed clear separation among all three groups, reinforcing the significant impact of both LPS challenge and probiotic intervention on hepatic metabolism (**Figure 7B**).

To identify potential biomarkers of ALI following *L. paracasei* FJG2337 intervention, we performed differential metabolite screening using volcano plot analysis comparing the model and FJG2337 groups. There were 109 key liver metabolites annotated between the model and FJG2337 groups (**Supplementary Figure S3**), including the down-regulation of 42 liver metabolites and the up-regulation of 67 liver metabolites (**Figure 7C**). Subsequently, pathway analysis was performed using the MetaboAnalyst platform with KEGG database annotation for the 109 identified metabolites (**Figure 7D**). KEGG enrichment analysis revealed significant involvement of these metabolites in nine major metabolic pathways: tryptophan metabolism, arachidonic acid metabolism, retinol metabolism, glutathione metabolism, linoleic acid metabolism, ascorbate and aldarate metabolism, pantothenate and CoA biosynthesis, vitamin B6 metabolism, and histidine metabolism.

### 3.7 Correlation analysis between gut microbiota, liver metabolites, and ALI-related biochemical parameters

Spearman correlation analysis was carried out to analysis relationships between ALI-related biochemical parameters, differential gut microbiota, differential liver metabolites regulated by *L. paracasei* FJG2337 treatment. The abundance of *Lacticaseibacillus*, *Lactobacillus* was positively associated with the concentration of cecal SCFAs (including butyric acid, valeric acid, isovaleric acid, acetic acid, isobutyric acid, and propionic acid), hepatic SOD, CAT, GSH, IL-10, but negatively associated with the level of hepatic IL-1β, IL-6, MDA, TNF-α (**Supplementary Figure S3**). However, the abundance of *Lachnospiraceae NK4A136 group*, *[Eubacterium]_ruminantium_group*, *norank_f_Lachnospiraceae*, *Lachnospiraceae UCG-006*, *Mucispirillum*, *Acetatifactor*, *BilophilaIntestinimonas*, *Bifidobacterium*, *Anaerofustis*, *Parasutterella* was positively associated with the concentration of serum ALP, serum ALT, serum AST, hepatic IL-6, hepatic TNF-α, hepatic MDA, but negatively associated with the concentration of cecal SCFAs, hepatic CAT, hepatic GSH, hepatic IL-10.

In addition, the relative concentration of sterebin B, Methyl 20-dihydroprednisolonate, acetdphenone, 9-oxoode, 9.10-Dihome, (+/-)13-Hpode, All-frans-18-Hydroxyretinoic acid, 1-Oleoylglycerophosphoserine, miglitol, sulfamethoxazole, nipecotic acid, 19(R)-Hydroxy-prostaglandin E1, elarofiban, thromboxane B2, eplerenone, vinylacetylene, solutol Hs 15, prostaglandin A2, 19(R)-Hydroxy-prostaglandin E2, erionic acid E, prostaglandin D2, 13-Hotre, 8-Iso-15-keto prostaglandin E2, prostaglandin K2, Fa(18:3 + 2O), (+/-)9-Hpode, prostaglandin E2, 2-hydroxypalmitic acid, (S)-10,16-dihydroxyhexadecanoic acid, raphanatin, leukotriene, indole-3-acetic acid, N6-methyladenosine, canrenone, lumichrome, 3-methylamino-1,2-propandiol, methenamine, Lps(20:4), Dn-isobutylamide, phenylalanyl-prolyl-arginine, (Z)-dodec-5-enedioic acid, myrsinoic acid B, 8,11-eicosadiynoic acid, 5-hydroxyindole-3-acetic acid, (E)-indol-3-ylacetaldoxime, fucoxanthinol, Poly Thf N6, 6-Ketoprostaglandin F1 Alpha, acetylcarnitine, 16-hydroxy-10-oxohexadecanoic acid, Dodecanedioic acid, dodecanedioic acid, dodecyl hydrogen sulfate, tetradecanediolc acid, 5-hydroxytryptophan, cyanidin 3-(6’’-acetyl-galactoside), brrabresib, plumieride, arg-asp-asp, methylimidazole acetaldehyde, tricarballylic acid, and val-pro-arg was positively associated with the relative abundance of *Lachnospiraceae_NK4A136_group*, *[Eubacterium]_ruminantium_group*, *norank_f_Lachnospiraceae*, *Lacticaseibacillus*, and *Lactobacillus*, but negatively associated with the relative abundance of norank_f-*Muribaculaceae*, *Bifidobacterium*, and *Dubosiella* (**Supplementary Figure S4**). However, the relative concentration of D-glucuronolactone, cysteinylglycine, L-glutathlone, fevipiprant, glu-gly-ser, taurocholenic acid, sulfolithocholylglycine, choline glycerophosphate, hallacridone, azinphos-ethyl, pantetheine 4’-phosphate, 4-methoxybenzyl O-(2-sulfoglucoside), Pe (O-10:0/8:0), Pe (20:0/0:0), Lpc(19:0-Sn1), Lpe(17:0), stearoyl lysophosphatidylethanolamine, Sm(D18:1/16:0), dimethyl phosphate, Lps(16:0), Sm(8:1_2O/26:0), Dg(Pgj2/1-22:0/0:0), L-A-lysophosphatidylserine, chikusetsusaponin la, Pe(Dime(11,5)/18:1(12Z0-O(9S, 10R)), Gpcho(16:0/18:3), pirprofen, orgothionenine, ergothioneine was negatively associated with the relative abundance of *Lachnospiraceae_NK4A136_group*, *[Eubacterium]_ruminantium_group*, *norank_f_Lachnospiraceae*, *Lacticaseibacillus*, and *Lactobacillus*, but positively associated with the relative abundance of *norank_f-Muribaculaceae*, *Bifidobacterium*, and *Dubosiella*.

## 4 Discussion

Growing evidence indicates that excessive oxidative-inflammatory responses play a crucial role in the pathogenesis of ALI (Qi et al., 2025; Xie et al., 2024). To investigate potential therapeutic interventions, an ALI mouse model was established through intraperitoneal LPS administration in this study. Our study demonstrates that *L. paracasei* FJG2337 pre-treatment attenuated LPS-induced elevation of serum hepatic enzymes (AST, ALT, and ALP), modulated GM composition, as well as improved hepatic metabolic function in ALI mice.

Serum AST and ALT levels serve as clinically established biomarkers for evaluating liver function, as these enzymes are primarily localized in hepatocyte mitochondria and cytoplasm (Qin et al., 2024). When the damage of hepatocyte, the hepatic AST and ALT were transferred to the blood, resulted to the increases in serum AST and ALT. Hepatocyte damage leads to the release of AST and ALT into circulation, resulting in elevated serum levels. In our study, LPS-treated mice showed significantly higher AST and ALT levels compared to controls, confirming successful establishment of the ALI model, consistent with previous findings (Dong et al., 2022). ALP, a membrane-bound hydrolase expressed in multiple tissues (particularly liver and bone), represents another important diagnostic marker for hepatic and skeletal disorders (Liu et al., 2017). Notably, *L. paracasei* FJG2337 pre-intervention remarkably attenuated LPS-induced elevations in all three hepatic enzymes (AST, ALT, and ALP), demonstrating its hepatoprotective potential against ALI.

Inflammation is strongly linked with oxidative stress in the liver, which promotes the onset and progression of ALI. TLR4 serve as one of the major receptors that plays a crucial role in inflammatory responses. Activated TLR4 elevates the mRNA expression of NF-кB, which stimulates the production of pro-inflammatory cytokines (including TNF-α, IL-1β and IL-6) (Lai et al., 2025). In this study, *L. paracasei* FJG2337 pre-intervention suppressed TNF-α, IL-1β and IL-6 concentrations in ALI mice, while elevated the hepatic IL-10 level. IL-10 is a key immunoregulatory cytokine that plays an essential role in improving liver function through activation of the JAK/STAT signaling pathway (Song et al., 2025). In addition, oxidative stress destroys the structure of protein, nuclear acids, and lipid in cells, which is one of the vital triggers in the pathogenesis of ALI (Lai et al., 2024). MDA destroy the enzyme activity on mitochondrial membranes, promoting hepatic lipid accumulation and hepatocyte necrosis by inhibiting fatty acid decomposition and inducing Mallory body accumulation in the cytoplasm (Tai et al., 2025). Nrf2 is an important transcription factor controlling the antioxidant response, and is strongly associated with the activity of antioxidant enzymes (SOD, GSH, and GSH-Px) (Li et al., 2025b). SOD is an antioxidant metalloenzyme existing in organisms, which catalyzes the disproportionation of superoxide anion radicals to product oxygen and hydrogen peroxide (Lou et al., 2024). Through the activation of GSH-Px and CAT, hydrogen peroxide is further catalyzed to water and oxygen, helping to eliminate reactive oxygen species accumulation (Milan et al., 2024). In this study, *L. paracasei* FJG2337 pre-intervention effectively enhanced hepatic SOD, GSH, and GSH-Px activities in ALI mice, while reducing hepatic MDA levels. Furthermore, up-regulation of Nrf2 expression promotes the expression of HO-1, one of the main cytoprotective antioxidants that suppresses leukocytic responses (Song et al., 2024). The hepatic Nrf2 and HO-1 expressions in ALI mice were significantly upregulated following *L. paracasei* FJG2337 pre-intervention. These results indicate that *L. paracasei* FJG2337 possesses anti-inflammatory and antioxidant properties by inhibiting the TLR4/NF-кB pathway and activating the Nrf2/HO-1 pathway, respectively.

Recently, “gut-liver axis” theory has received widespread attention worldwide, and is regarded as a vital factor in improving and treating liver function-related diseases (Ray, 2024). In this study, the proportions of *Mucispirillum*, *Lachnospiraceae_NK4A136_group*, *Anaerotruncus*, and *Acetatifactor* in the model group were higher than those in the control group. *Mucispirillum*, a member of the phylum Deferribacteres, is widely distributed in the intestinal tracts of humans and animals, and promotes inflammatory responses by inducing LPS production (Herp et al., 2021). *Lachnospiraceae_NK4A136_group* is a controversial bacterium that has been reported to be positively associated with liver function-related diseases (Xu et al., 2024). *Anaerotruncus*, a Gram-positive anaerobic bacterium, has been documented to impair liver function by disrupting glucolipid metabolism and stimulating inflammatory responses (Su et al., 2020). *Acetatifactor* is prevalent in the gut microbiota of patients with obesity and diabetes, and has been shown to promote pro-inflammatory cytokine secretion through LPS accumulation, thereby exacerbating liver dysfunction. Several studies have confirmed that probiotics can improve liver function by modulating GM composition (Xu et al., 2023). Our study found that *L. paracasei* FJG2337 pre-intervention remarkably elevated the proportions of *Lactobacillus* and *Lacticaseibacillus* in ALI mice. These represent classic lactic acid bacteria strains commonly found in the gut that play crucial roles in maintaining host health, such as the regulation of bile acids, modulates GM disorder, and mitigates hepatocyte apoptosis (Wu et al., 2025). *Lactobacillus* demonstrates antioxidant and anti-inflammatory functions by both secreting a series of antioxidant enzymes and inhibiting the TLR4/NF-κB pathway (Cai et al., 2025). A previous study showed that *Lactobacillus* intervention elevates the intestinal indole derivatives by tryptophan metabolism, which help to improve the host’s immune system (Xia et al., 2023). Moreover, the proportion of *Lactobacillus, Lacticaseibacillus*, and the concentration of hepatic indole derivatives (including indole-3-acetic acid, indol-3-acetamide, 5-hydroxyindole-3-acetic acid, (E)-indol-3-ylacetaldoxime), cecal SCFAs (including acetic acid, valeric acid, butyric acid, iso-butyric acid) in the FJG2339 group was remarkably higher than that in the model group, indicating *L. paracasei* FJG2339 successfully colonized the intestinal tract and produced some indole derivatives that prevent LPS from damaging the liver. In addition, *Lacticaseibacillus* is a common SCFAs-producing bacterium in the intestine, not only maintain intestinal barrier integrity by providing energy to intestinal epithelial cells, and enhance the host immunity system by regulating immune cells (Junior et al., 2025). Interestingly, *L. paracasei* FJG2337 pre-treatment significantly decreased the proportions of *Parasutterella*, *Bilophila*, and *Bifidobacterium*. *Parasutterella*, a relatively new genus, accelerates hepatic accumulation of fatty acid and stimulates the inflammatory responses in the gut (Li et al., 2025a). *Bilophila* is a widespread intestinal pathobiont that promotes the conversion of taurine and isethionate (originated from taurine-conjugated bile salts) into hydrogen sulfide, which damages gut integrity and facilitates the entry of harmful substances into circulation (Liu et al., 2023). However, *Bifidobacterium* is a beneficial gut bacterium that ferments carbohydrates to produce SCFAs and other advantageous metabolites, helping to maintain gut barrier integrity and enhance host immunity (Shan et al., 2025). Interestingly, the abundance of *Bifidobacterium* in the FJG2339 group were lower than that in the model, possibly due to competitive exclusion by *Lactobacillus*, which may suppress *Bifidobacterium* growth via acid production and nutrient competition. These results suggest that *L. paracasei* FJG2337 can modulate gut microbiota composition by increasing beneficial bacteria, potentially contributing to improved liver function.

To further elucidate the potential mechanism of *L. paracasei* FJG2337 pretreatment in LPS-induced ALI mice, liver metabolomics were implemented in this study. In the present study, the hepatic myrsinoic acid B, indole-3-acetic acid, indole-3-acetamide, D-glucuronolactone, sulfamethoxazole, and 4-pyridoxic acid in ALI mice following *L. paracasei* FJG2337 pre-treatment. Among these, myrsinoic acid B has been reported to exhibit multiple biological activities, including anti-inflammatory, antimicrobial, and antitumorigenic properties (dos Santos et al., 2025). Yang et al. demonstrated that myrsinoic acid B effectively suppresses LPS-induced inflammatory responses by regulating both Src/Syk/NF-κB and IRAK-1/AP-1 pathways (Yang et al., 2014). Indole-3-acetic acid and indole-3-acetamide, as microbiota-derived metabolites, inhibit TLR4 expression while enhancing hepatic GSH and SOD activity, thereby preventing ALI onset and progression (Aljarboa et al., 2023; Wang et al., 2023). D-Glucuronolactone, naturally abundant in animal livers, demonstrates hepatoprotective effects by enhancing the activity of antioxidant enzymes (SOD, GPx and GSH) and decreasing the production of inflammatory cytokines (IL-1β, IL-6 and TNF-α) in thioacetamide-induced liver fibrosis models (Chen et al., 2015). Sulfamethoxazole, a synthetic organic compound, mitigates ALI progression by enhancing SOD, GSSG, TAO, and GSH activity (Zahran et al., 2020). 4-Pyridoxic acid, a vitamin B6 derivative, helps ameliorate hepatic and renal damage (Wang et al., 2023). However, the concentration of hepatic azinphos-ethyl, pirprofen, N-acetyltaurine, and lysophosphatidylserine in ALI mice after *L. paracasei* FJG2337 treatment. Azinphos-ethyl is a heterocyclic organophosphorus compound that destroy the live function and digestive system. Pirprofen is a non-steroidal anti-inflammatory drug, while long-time consumption of pirprofen promote the accumulation of fatty acid by suppressing the mitochondrial beta-oxidation (Geneve et al., 1987). N-acetyltaurine is one of the acetylation products of taurine that widely used as the ethanol marker in human blood, and high concentration of N-acetyltaurine induced the injury of liver function (Luginbühl et al., 2017). Lysophosphatidylserine is a deacylated form of phosphatidylserine that stimulate the occurrence of liver fibrosis by regulating the inflammatory responses in the tissue neutrophilia (Nishikawa et al., 2022). Based on these results, it can be speculated that alters in liver metabolites take an essential role in improving liver inflammatory responses and oxidative stress induced by LPS, highlighting the underlying therapeutic benefits of *L. paracasei* FJG2337 in alleviating ALI.

## 5 Conclusion

Our findings demonstrate that *L. paracasei* FJG2337 pretreatment ameliorated ALI through reducing hepatic pro-inflammatory cytokines via TLR4/NF-κB pathway suppression and enhancing antioxidant enzymes through Nrf2/HO-1 pathway activation. In addition, *L. paracasei* FJG2337 pre-intervention regulated the GM composition and improved hepatic metabolism in ALI mice. However, the exact GM-liver function relationships require validation through fecal microbiota transplantation, and the key metabolites involved in these interactions need further confirmation.

## Data Availability

The datasets presented in this study can be found in online repositories. The names of the repository/repositories and accession number(s) can be found below: https://www.ncbi.nlm.nih.gov/, PRJNA1122694.
