# Supplementary material for: Lacticaseibacillus paracasei FJG2337 mitigate acute liver injury-related inflammatory responses, gut microbiota and liver metabolism in mice
Source: Front Cell Infect Microbiol. 2025 Oct 21;15:1674551. doi: 10.3389/fcimb.2025.1674551 (PMC12582951; doi:10.3389/fcimb.2025.1674551)
Supplement: Supplementary file 1 [file Table1.docx]

**Supplementary materials**

**Figure S1** The free radical scavenging ability of four strains of *L. paracasei* *in vivo*


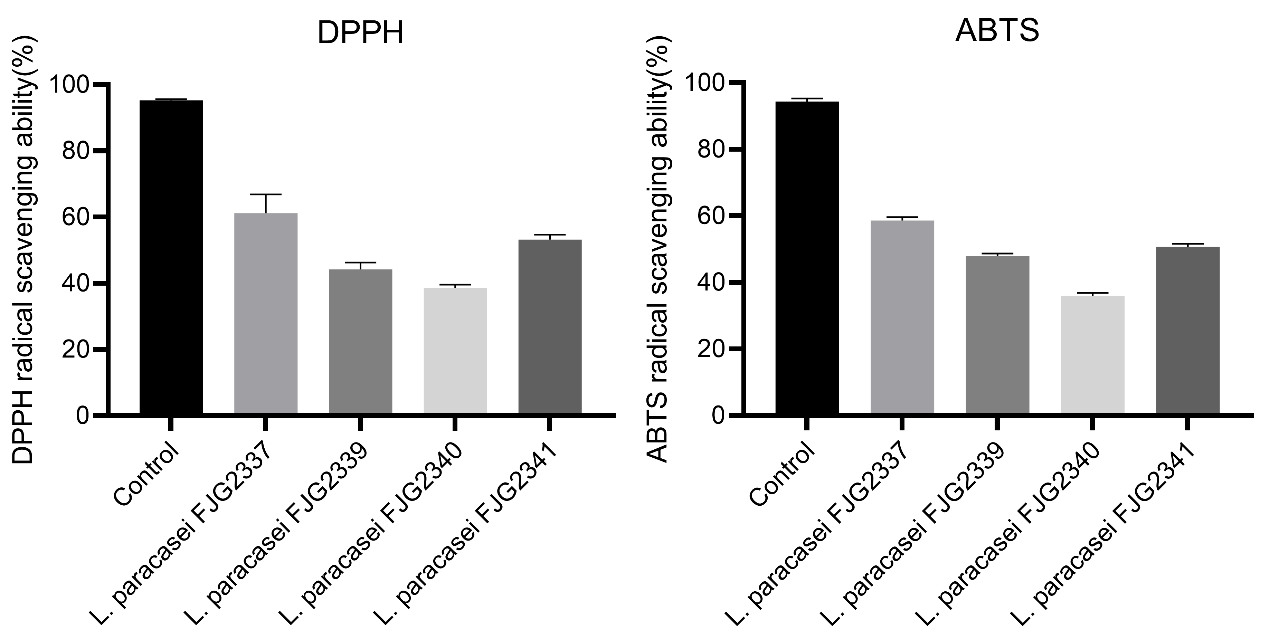


**Figure S2** Volcano plot screened differential metabolites between the FJG2337 and model groups


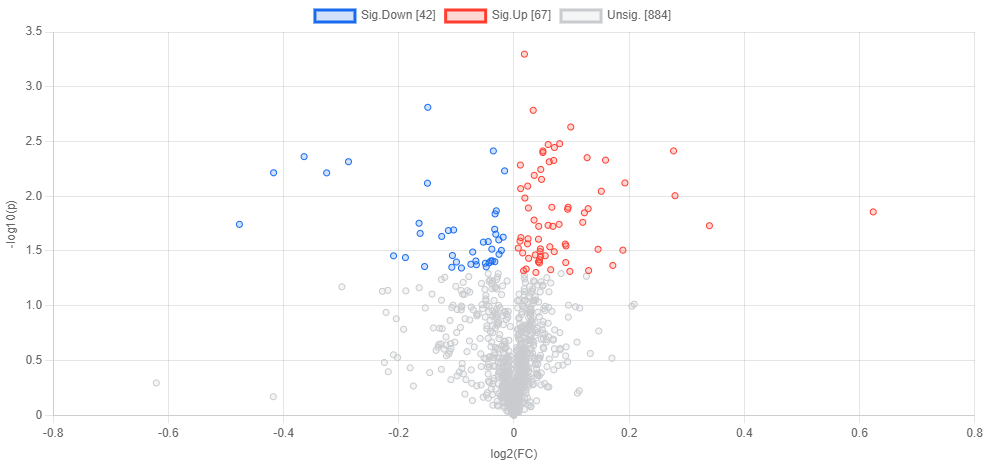


**Figure S3** Heatmap of the correlation analysis among key gut microbiota and ALI-related biochemical parameters.


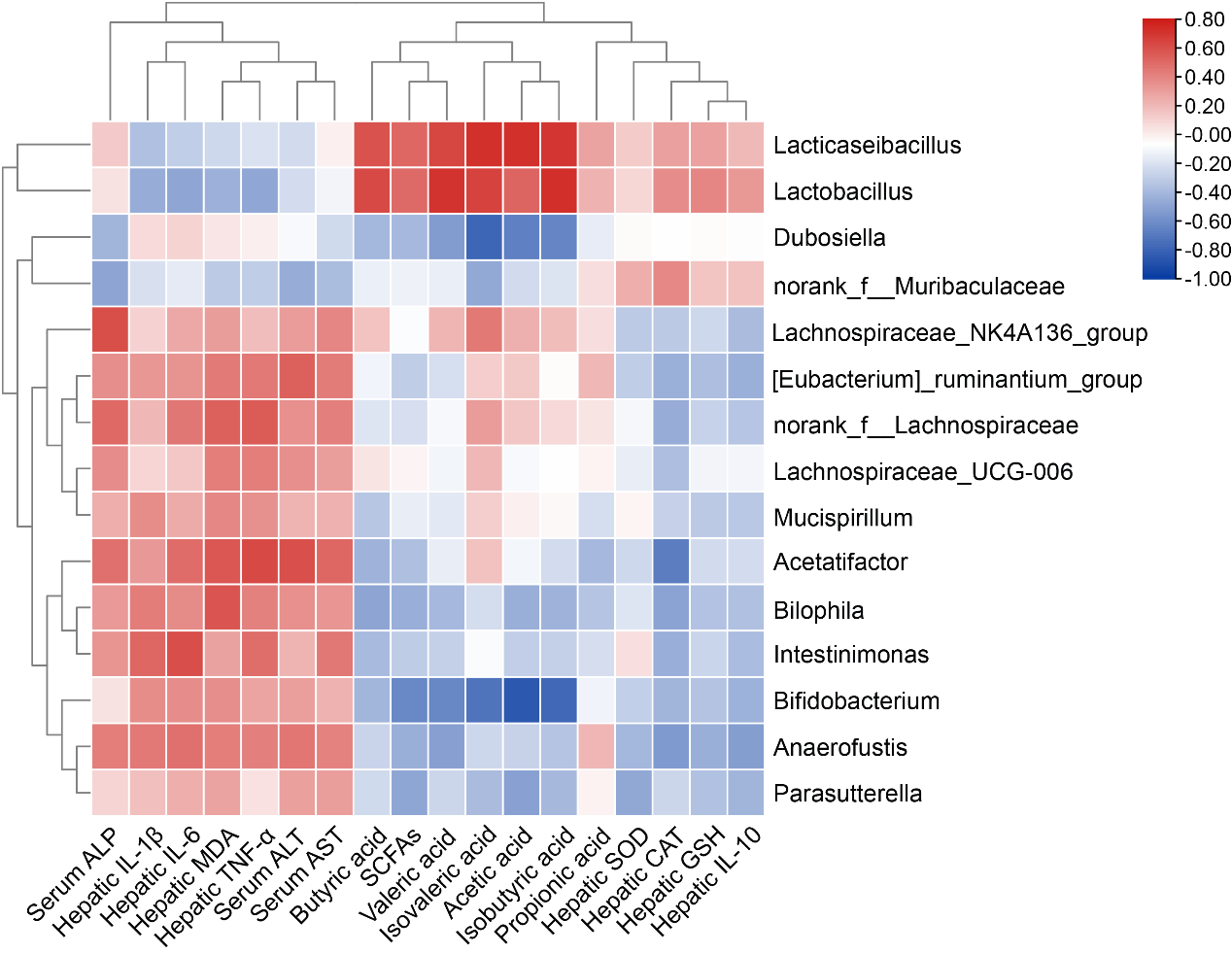


**Figure S4** Heatmap of the correlation analysis among key liver metabolites and key gut microbiota or ALI-related biochemical parameters.


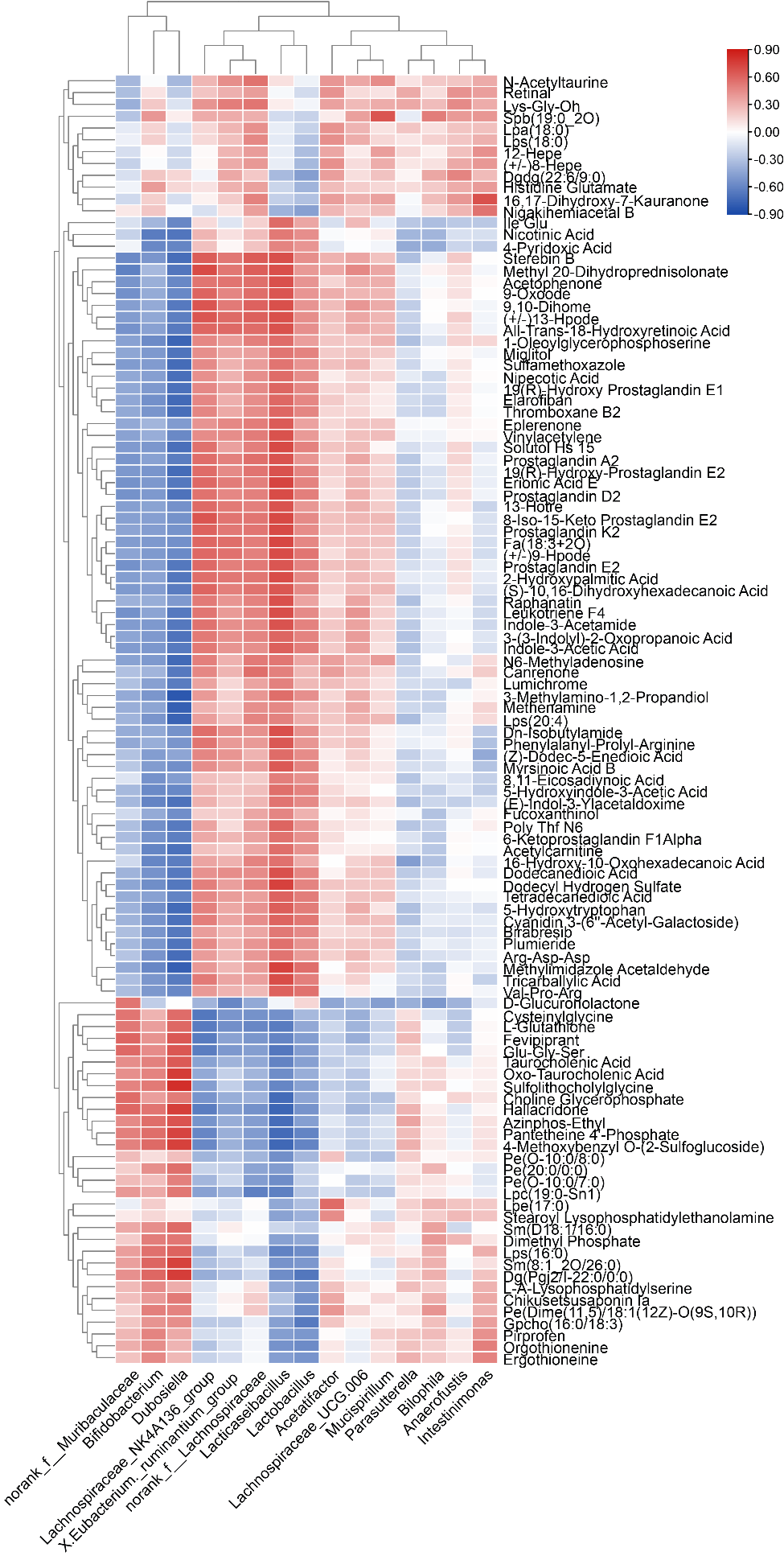


**Table S1** Primer pairs for Real-time Quantitative PCR analysis of indicated genes.

| Gene | Forward sequence (5’-3’) | Reserve sequence (3’-5’) |
| --- | --- | --- |
| TLR4 | GGCAGCAGGTGGAATTGTAT | AGGCCCCAGAGTTTTGTTCT |
| MyD88 | GTGCCGTCGGATGGTAGTG | GACAGTGATGAACCGCAGGAT |
| NF-кB | CGCCCCTTATCGACCACC | CCTTCTCCCAAGAGTCGTCCA |
| Iк-Bα | ACCAACCAGCCAGAAATCG | TCACAGGCAAGGTGTAGAGGG |
| Cox2 | GGGAGTCTGGAACATTGTGAA | GCACGTTGATTGTAGGTGGACTGT |
| iNOS | CTTGGAGCGAGTTGTGGATTGTC | TAGGTGAGGGCTTGGCTGAGTG |
| HO-1 | ACATCCAAGCCGAGAATGCTG | CCAGTGAGGCCCATACCAGA |
| Arg1 | AGACAGCAGAGGAGGTGAAGA | CGAAGCAAGCCAAGGTTAAAG |
| β-Actin | TGGAATCCTGTGGCATCCATGAAAC | TAAAACGCAGCTCAGTAACAGTCCG |

**Table S2** Key gut microbiota abundance between the model and control or FJG2337 groups.

| Name | Control | Model | *p* value |
| --- | --- | --- | --- |
| *norank_f__Muribaculaceae* | 51.81±6.74 | 41.47±4.60 | <0.01 |
| *Lachnospiraceae_NK4A136_group* | 4.61±1.97 | 4.49±3.77 | 0.02 |
| *Mucispirillum* | 0.65±0.66 | 1.49±0.99 | 0.04 |
| *norank_f__Lachnospiraceae* | 0.30±0.17 | 0.81±0.33 | <0.01 |
| *Anaerotruncus* | 0.27±0.10 | 0.54±0.25 | 0.02 |
| *[Eubacterium]_ruminantium_group* | 0.07±0.14 | 0.56±0.41 | <0.01 |
| *Acetatifactor* | 0.09±0.07 | 0.28±0.17 | 0.01 |
| *Lachnospiraceae_UCG-006* | 0.08±0.07 | 0.28±0.17 | 0.04 |
| Name | FJG2337 | Model | *p* value |
| *Lactobacillus* | 8.65±3.88 | 1.83±0.68 | <0.01 |
| *Dubosiella* | 0.47±0.23 | 2.67±3.58 | <0.01 |
| *Bifidobacterium* | 0.07±0.08 | 1.39±1.29 | <0.01 |
| *Parasutterella* | 0.28±0.28 | 0.54±0.33 | 0.04 |
| *Bilophila* | 0.18±0.08 | 0.64±0.40 | <0.01 |
| *Acetatifactor* | 0.12±0.09 | 0.28±0.17 | 0.04 |
| *Lacticaseibacillus* | 0.33±0.22 | 0±0 | <0.01 |
| *Intestinimonas* | 0.06±0.05 | 0.14±0.07 | 0.02 |

**Table S3** The fold changes in key liver metabolites abundance between the model and FJG2337 groups.

| Down-regulation of liver metabolites (FJG2337 Vs. model) | | | |
| --- | --- | --- | --- |
| Name | FC | Name | FC |
| Fevipiprant | 0.72 | Spb(19:0_2O) | 0.96 |
| Cysteinylglycine | 0.75 | Pe(Dime(11,5)/18:1(12Z)-O(9S,10R)) | 0.96 |
| Azinphos-Ethyl | 0.78 | Lpe(17:0) | 0.96 |
| 4-Methoxybenzyl O-(2-Sulfoglucoside) | 0.80 | Retinal | 0.97 |
| Pantetheine 4'-Phosphate | 0.82 | Pe(O-10:0/8:0) | 0.97 |
| Lys-Gly-Oh | 0.87 | (+/-)8-Hepe | 0.97 |
| Pirprofen | 0.88 | Nigakihemiacetal B | 0.97 |
| L-Glutathione | 0.89 | 12-Hepe | 0.97 |
| Sulfolithocholylglycine | 0.89 | Pe(O-10:0/7:0) | 0.97 |
| Sm(D18:1/16:0) | 0.90 | Gpcho(16:0/18:3) | 0.97 |
| Glu-Gly-Ser | 0.90 | Dgdg(22:6/9:0) | 0.98 |
| Hallacridone | 0.90 | Choline Glycerophosphate | 0.98 |
| Orgothionenine | 0.92 | Stearoyl Lysophosphatidylethanolamine | 0.98 |
| Sm(8:1_2O/26:0) | 0.92 | N-Acetyltaurine | 0.98 |
| Chikusetsusaponin Ia | 0.93 | Taurocholenic Acid | 0.98 |
| 16,17-Dihydroxy-7-Kauranone | 0.92871 | Lpa(18:0) | 0.98 |
| Ergothioneine | 0.93 | L-A-Lysophosphatidylserine | 0.98 |
| Lpc(19:0-Sn1) | 0.93 | Pe(20:0/0:0) | 0.98 |
| Oxo-Taurocholenic Acid | 0.94 | Lps(18:0) | 0.99 |
| Histidine Glutamate | 0.95 | Dimethyl Phosphate | 0.99 |
| Dg(Pgj2/I-22:0/0:0) | 0.95 | Lps(16:0) | 0.99 |
| Up-regulation of liver metabolites (FJG2337 Vs. model) | | | |
| Name | FC | Name | FC |
| Lps(20:4) | 1.01 | Prostaglandin D2 | 1.04 |
| Methenamine | 1.01 | 19(R)-Hydroxy Prostaglandin E1 | 1.04 |
| 3-Methylamino-1,2-Propandiol | 1.01 | 6-Ketoprostaglandin F1Alpha | 1.04 |
| Nipecotic Acid | 1.01 | 13-Hotre | 1.05 |
| 1-Oleoylglycerophosphoserine | 1.01 | Prostaglandin A2 | 1.05 |
| Fucoxanthinol | 1.01 | Prostaglandin E2 | 1.05 |
| 8,11-Eicosadiynoic Acid | 1.01 | Elarofiban | 1.05 |
| Methylimidazole Acetaldehyde | 1.01 | Miglitol | 1.05 |
| Acetylcarnitine | 1.01 | Val-Pro-Arg | 1.05 |
| N6-Methyladenosine | 1.01 | Plumieride | 1.06 |
| Lumichrome | 1.02 | 19(R)-Hydroxy-Prostaglandin E2 | 1.06 |
| Arg-Asp-Asp | 1.02 | Fa(18:3+2O) | 1.06 |
| Ile Glu | 1.02 | (E)-Indol-3-Ylacetaldoxime | 1.06 |
| Canrenone | 1.02 | (Z)-Dodec-5-Enedioic Acid | 1.06 |
| 2-Hydroxypalmitic Acid | 1.02 | Birabresib | 1.07 |
| Poly Thf N6 | 1.02 | 8-Iso-15-Keto Prostaglandin E2 | 1.07 |
| 9,10-Dihome | 1.02 | Methyl 20-Dihydroprednisolonate | 1.07 |
| Dodecyl Hydrogen Sulfate | 1.02 | Erionic Acid E | 1.07 |
| Vinylacetylene | 1.03 | Leukotriene F4 | 1.09 |
| Eplerenone | 1.03 | 9-Oxoode | 1.09 |
| Solutol Hs 15 | 1.03 | Indole-3-Acetamide | 1.09 |
| Acetophenone | 1.03 | Prostaglandin K2 | 1.09 |
| Tetradecanedioic Acid | 1.03 | Sulfamethoxazole | 1.09 |
| 16-Hydroxy-10-Oxohexadecanoic Acid | 1.03 | Raphanatin | 1.11 |
| Nicotinic Acid | 1.03 | All-Trans-18-Hydroxyretinoic Acid | 1.11 |
| (+/-)13-Hpode | 1.03 | Cyanidin 3-(6''-Acetyl-Galactoside) | 1.12 |
| (S)-10,16-Dihydroxyhexadecanoic Acid | 1.03 | D-Glucuronolactone | 1.13 |
| 4-Pyridoxic Acid | 1.03 | 5-Hydroxyindole-3-Acetic Acid | 1.14 |
| Dodecanedioic Acid | 1.03 | Indole-3-Acetic Acid | 1.14 |
| Thromboxane B2 | 1.03 | Tricarballylic Acid | 1.21 |
| 3-(3-Indolyl)-2-Oxopropanoic Acid | 1.04 | Myrsinoic Acid B | 1.21 |
| 5-Hydroxytryptophan | 1.04 | Dn-Isobutylamide | 1.27 |
| (+/-)9-Hpode | 1.04 | Phenylalanyl-Prolyl-Arginine | 1.54 |
| Sterebin B | 1.04 |  |  |
